# Genomic Characterization of a Class 2/1 Hybrid Integron-Driven Multidrug-Resistant *Proteus mirabilis* SH-8 of Swine Origin

**DOI:** 10.3390/vetsci12121113

**Published:** 2025-11-22

**Authors:** Xinyu Wang, Hui Su, Nuo Xu, Donghui Tang, Sibo Liu, Jiarui Lin, Wanli Sha, Baishuang Yin, Wenlong Dong

**Affiliations:** 1 College of Animal Science and Technology, Jilin Agricultural Science and Technology University, 77 Hanlin Road, Jilin 132101, China; 18644919202@163.com (X.W.);; 2 Jilin Provincial Key Laboratory of Preventive Veterinary Medicine, Jilin 132101, China; 3 Jilin Province Technology Innovation Center of Pig Ecological Breeding and Disease Prevention and Control, Jilin 132101, China; 4 Jilin Province Cross Regional Cooperation Technology Innovation Center of Porcine Main Disease Prevention and Control, Jilin 132101, China

**Keywords:** *Proteus mirabilis*, multidrug resistance, whole-genome sequencing, integron

## Abstract

**Objective:** *P. mirabilis* is a Gram-negative rod-shaped bacterium. In this study, the SH-8 strain of *P. mirabilis* was isolated from swine-derived samples, and its antibiotic resistance genes (ARGs) and transmission mechanisms were systematically characterized. **Methods:** The minimum inhibitory concentrations (MICs) of strain SH-8 were determined by the broth microdilution method, with genomic features analyzed through whole-genome sequencing (WGS). **Results:** *P. mirabilis* SH-8 exhibited resistance to multiple antimicrobial agents. Its genome consists of a single chromosome (3,993,987 bp; GC content 38.8%) and one plasmid. WGS identified 21 distinct ARGs in this strain, along with a class 2/1 hybrid integron. Furthermore, a heatmap analysis of resistance genes from 29 *P. mirabilis* isolates of diverse origins revealed a striking similarity between the resistance gene profile of SH-8 and those of other strains. **Conclusions:** The MDR exhibited by *P. mirabilis* SH-8 is primarily attributed to integron-mediated resistance gene clusters within its genome, which may pose challenges for treating opportunistic infections caused by this bacterium.

## Simple Summary

*Proteus mirabilis* (*P. mirabilis*), a Gram-negative bacterium of the *Enterobacteriaceae* family, is commonly found as part of the normal intestinal flora of pigs, yet it also possesses the potential to act as an opportunistic pathogen capable of causing infections in both humans and animals. This strain was found to harbor multiple antibiotic resistance genes (ARGs) and a hybrid integron of classes 2 and 1, highlighting its complex drug resistance mechanisms. These findings confirm the significant role of integrons in the dissemination of resistance genes.

## 1. Introduction

*Proteus* spp. are Gram-negative, non-sporulating, non-encapsulated bacilli within the family *Enterobacteriaceae*. The genus comprises five validated species: *P. mirabilis*, *Proteus hauseri*, *Proteus myxofaciens*, *Proteus vulgaris*, and *Proteus penneri*. Among them, P. mirabilis has the potential to cause opportunistic infections in both humans and animals, and is of significant clinical relevance. Key virulence genes in *P. mirabilis* include those encoding fimbrial proteins such as *mrpA*, *pmfA*, *atfA*, and *ucaA*; proteases such as *zapA* and *ptA*; hemolysins including *hpmA* and *hlyA*; and the siderophore receptor gene *ireA* [1]. As an opportunistic pathogen, it is the second most common pathogen causing urinary tract infections in humans and animals, second only to *Escherichia coli* (*E. coli*) [2]. The pathogenicity of *P. mirabilis* in the urinary tract is attributed to multiple virulence factors, including motility, urease production, hemolysin secretion, biofilm formation, and fimbriae-mediated adhesion [3]. As a result, once *P. mirabilis* colonizes the urinary tract, it can cause difficult-to-treat infections or catheter obstruction [4]. This bacterium demonstrates ubiquitous environmental distribution, inhabiting soil, aquatic systems, and wastewater, while also establishing commensal colonization in the gastrointestinal tracts of mammalian hosts. *P*. *mirabilis* can infect a wide range of wild animals and is also commonly found in domestic livestock such as cattle, sheep, chickens, and pigs. As pork is a major meat source in China, preventing *P. mirabilis* opportunistic infection in pigs is of significant importance. According to Qu et al., the bacterium was detected in 5.55% (30/541) of pig samples in Zhejiang Province, with the highest detection rate observed in the Jinhua region [5]. According to Ge et al., in Guangxi Zhuang Autonomous Region, the bacterium was detected in 21.42% (21/98) of fecal and tissue samples from diseased pigs, suggesting its potential pathogenicity [6]. Opportunistic infection of *P*. *mirabilis* can lead to urinary tract infections, septicemia, or respiratory infections in pigs.

Notably, *P. mirabilis* exhibits intrinsic resistance to multiple antimicrobial classes including nitrofurantoin, tetracyclines (tigecycline), and polymyxins [7]. The escalating global prevalence of MDR strains across North America [8], South America [9], Europe [10], Africa [11], and Asia [12] has emerged as a pressing public health concern; in some countries, strains harboring extended-spectrum β-lactamases (ESBLs) or AmpC-type cephalosporinases have disseminated widely, demonstrating co-resistance to a broad range of anti-infective drugs beyond their core resistance to penicillins and cephalosporins (including oximino-cephalosporins) [13,14,15,16,17]. The rise in (MDR) bacterial infections poses a critical challenge to global public health, imposing severe morbidity and mortality burdens worldwide. Current projections suggest antimicrobial resistance (AMR) could directly claim 1.91 million lives annually by 2050 [18]. Bacterial acquisition of AMR occurs via two primary mechanisms, (1) genomic mutations and (2) horizontal gene transfer (HGT), with the latter predominantly mediated by mobile genetic elements (MGEs). MGEs comprise diverse elements including insertion sequences (IS), transposons, bacteriophages, plasmids, genomic islands, and integrative conjugative elements (ICEs) [19]. Among these, plasmids—extrachromosomal, self-replicating DNA molecules—are of particular concern due to their role as principal vectors for ARGs. These elements drive resistance dissemination by facilitating inter- and intra-species transfer of ARGs through conjugation, transformation, or transduction.

This study aimed to analyze the resistance phenotype and resistance gene profiles of a swine-derived *P. mirabilis* isolate, SH-8, with the objective of gaining deeper insights into the potential risks associated with these MDR *P. mirabilis* isolates.

## 2. Materials and Methods

### 2.1. Isolation and Identification of Bacteria

In this study, a nasal swab was collected from a diseased pig with respiratory symptoms on a small-scale farm in Jilin Province, China, and inoculated onto Brain Heart Infusion (BHI) agar for culture. After incubation at 37 °C for 12 h, single colonies were isolated and purified. DNA extraction of bacterial isolates was carried out using a Bacterial Genomic Isolation Kit (Sangon Biotech Shanghai China) according to the manufacturers instructions and to Comate Bioscience Co., Ltd. (Changchun, China) confirmed by 16SrRNA sequencing.

### 2.2. Antimicrobial Susceptibility Testing

Additionally, the minimum inhibitory concentration (MIC) of SH-8 was determined by broth microdilution, including: amoxicillin, ampicillin, cefazolin, ceftriaxone, aztreonam, azithromycin, chloramphenicol, trimethoprim, gentamicin. The bacterial suspension was adjusted to a concentration of 1 × 10^7^ CFU/mL, and then subjected to dilution in broth medium, yielding a final inoculum concentration of approximately 5 × 10^5^ CFU/mL per well. Antibiotic concentrations ranged from 0.03 to 512 μg/mL. The procedure followed the Clinical and Laboratory Standards Institute (CLSI) 2025 guidelines (33rd edition). The plates were incubated at 37 °C for 16 h, after which the results were recorded. *E. coli* ATCC 25922 served as the quality control strain.

### 2.3. Whole Genome Sequencing (WGS)

The WGS of *P. mirabilis* SH-8 was performed by Biomarker Technologies (Beijing, China). The Canu v1.5 package was then used to assemble the filtered data, and the quality of the assembly was double checked using Racon v3.4.3 software and Circlator v1.5.5 software with default. ARGs of *P. mirabilis* SH-8 was identified using the ResFinder database with CARD BLAST and RGI (https://card.mcmaster.ca/analyze/rgi Accessed on 23 May 2023) and screened for resistance genes according to strict and perfect. GCView [20] and Snapgene were used to visualize the genomic structure of the whole chromosome and one plasmid.

### 2.4. Nucleotide Sequence Accession Number

The complete genome sequence of *P. mirabilis* SH-8 was deposited at GenBank databases under the accession number CP181087. And one plasmids, was submitted to GenBank under accession number CP181088.

## 3. Results

### 3.1. SH-8 Identification Result

The colony morphology of strain SH-8 on solid medium is circular and filamentous, demonstrating spreading growth and chemotaxis. Strain SH-8 was identified as *P. mirabilis* based on 16S rRNA gene sequencing. Analysis performed with the National Center for Biotechnology Information (NCBI) BLAST tool confirmed 99% sequence identity.

### 3.2. Antibiotic Resistance Profiles of P. mirabilis SH-8

According to the CLSI 2025 guidelines, antimicrobial susceptibility testing of the *P. mirabilis* SH-8 strain was performed using the broth microdilution. Using the MIC interpretive criteria for *Enterobacteriaceae*, the strain was resistant to five antimicrobial agents. The specific breakpoints and susceptibility results are shown in Table 1.

### 3.3. Analysis of ARGs in P. mirabilis SH-8

The complete genome sequences of *P. mirabilis* SH-8 was assembled. SH-8 has a 3,993,987 bp chromosome and carries one plasmid (44,609 bp). All identified antibiotic ARGs were chromosomally located. Through BLAST alignment against the Comprehensive Antibiotic Resistance Database (CARD), we detected 21 ARGs in *P. mirabilis* SH-8: *sul2*, *aph*(*4)-Ia*, *aac(3)-IVa*, *sul1*, *qacEdelta1*, *aadA*, *msrE*, *mphE*, *blaDHA-1*, *aac(6′)-Ib10*, *dfrA1*, *lnuF*, *rsmA*, *catA4*, *blaCRP*, and *cfrA*.

### 3.4. Genomic Analysis of Strains Based on Mobile Genetic Elements

**Figure 1:**
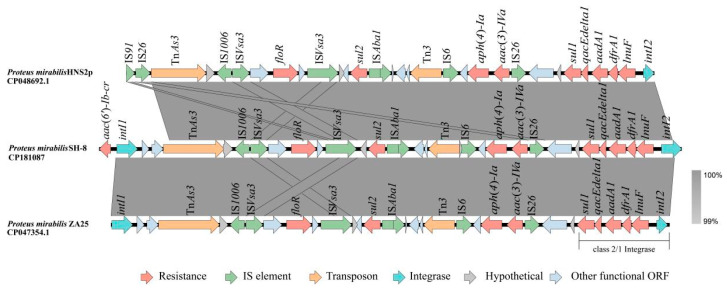
Genetic structure of class 2 integrons associated with *P. mirabilis* SH-8. The sequences used for charting are linked to the following GenBank accession numbers HNS2p (*P. mirabilis* CP048692.1), ZA25 (*P. mirabilis* CP047354.1). Genes and ORFs are shown as arrows, and the direction of transcription is indicated by arrows. Shared areas with 99% identity will be colored. Resistance, transposon, and integrase genes are shown in red, orange, and cyan, respectively.

A comparison of gene clusters showed that a hybrid class 2/class 1 integron, containing the gene cassette array *sull-qacEdelta1-aadA1-dfrA1-lnuF-intI2*, is present in *P. mirabilis* SH-8 (Figure 1). This hybrid structure likely originated from homologous recombination between the conserved regions of class 2 and class 1 integrons. Notably, *P. mirabilis* SH-8 shares an identical class 2/1 integron structure with *P. mirabilis* HNS2P and *P. mirabilis* ZA25. This genetic similarity could suggest clonal spread of a successful bacterial strain. Alternatively, it may indicate that these integrons, as mobile genetic elements, have been disseminated among different *P. mirabilis* lineages through horizontal gene transfer (HGT) mechanisms such as conjugation, transformation, or transduction.

Compare gene clusters analysis showed 99% nucleotide identity and 99% coverage between *P. mirabilis* SH-8 and the two other strains (ZA25 and HNS2P). This high genetic similarity suggests that frequent interregional animal trade may have facilitated the dissemination of these resistance genes, leading to nearly identical resistance gene structures in *P. mirabilis* strains isolated from three distinct geographic regions.

### 3.5. Heat Map Analysis

The heatmap illustrates the distribution patterns of genotypic and phenotypic characteristics between SH-8 and other strains, aiming to explore potential links among the phenotypic trait profiles of strains from diverse sources.

**Figure 2:**
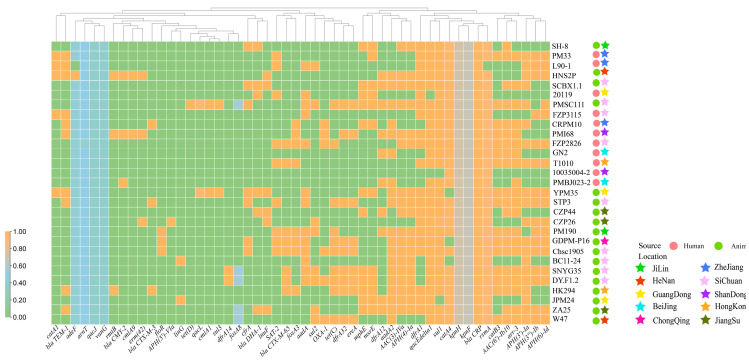
Heatmap of ARGs in *P. mirabilis* from diverse sources. The figure presents a heatmap of ARGs in SH-8 and other *P. mirabilis* strains from diverse sources, illustrating the differences and similarities in resistance gene profiles among these isolates. Sample annotations include: collection location, isolation source, and ARGs. In the heatmap, orange indicates the presence of a gene, while green denotes its absence. The heatmap was generated using TBtools-II (Toolbox for Biologists v2.357) software.

We selected 29 *P. mirabilis* strains from diverse geographical regions and sources (xlsx S1), and constructed a heatmap to visualize their antibiotic resistance genotypes. Analysis of the heatmap revealed that the resistance gene profiles of other *P. mirabilis* strains were highly similar to that of SH-8 (Figure 2). Of particular note, the resistance gene profiles were highly congruent between human and animal *P. mirabilis* isolates in the heatmap, suggesting active cross-species transmission of this zoonotic pathogen. Additionally, five genes—*rsmA*, *blaCRP*, *catA4*, *sul1*, and *qacEdelta1*—were common to all *P. mirabilis* strains tested (Figure 2). The widespread presence of these resistance genes across the strains suggests that they may constitute part of the core genome of *P. mirabilis*, playing a critical role in the adaptation and survival of this bacterium in diverse environments. Additionally, it was observed that the resistance gene *catA1*, carried by SH-8, was rarely detected in other strains.

## 4. Discussion

In recent years, there have been increasing reports of *P*. *mirabilis* being isolated from animals [21,22]. As pigs are one of the most important livestock species in animal farming, *P. mirabilis* opportunistic infection can lead to substantial economic losses for the industry. Research shows that *P. mirabilis* strains isolated from pigs exhibit multidrug resistance, underscoring the importance of research on swine-origin *P. mirabilis*. In this study, *P. mirabilis* strain SH-8 was isolated from nasal swabs of diseased pigs on farms in Jilin Province. The *P. mirabilis* strain SH-8 carries a classic class 2 integron. Integrons are mobile genetic elements capable of integrating and expressing exogenous gene cassettes. According to Moreira A et al., in *P. mirabilis* isolates, class 1 integron was less common, with a detection rate of 13.8% (45/327). In contrast, class 2 integron was more prevalent at 31.5% (103/327), suggesting a stronger link to this species [23].

Integrons can cascade multiple distinct resistance genes, thereby promoting the formation of MDR, horizontal transfer of ARGs enables bacteria to rapidly acquire diverse resistance mechanisms, leading to the emergence of MDR, extensively drug-resistant (XDR), and even pan-drug-resistant (PDR) strains. These resistance genes are disseminated across different bacterial species, genera, and even kingdoms via mobile genetic elements (MGEs) such as plasmids, transposons, and integrons, thereby vastly expanding the proliferation of AMR. We identified two instances in SH-8 where the antimicrobial susceptibility testing results did not match the expected genotype. First, SH-8 is sensitive to chloramphenicol despite carrying the associated resistance gene *catA4*. Since the sequence identity of *catA4* in CARD Blast is only 91.63%, this discrepancy may be due to the gene being incomplete and thus unable to produce a functional protein. Secondly, although it carries the *blaDHA* gene, it remains susceptible to penicillin drugs. This is likely due to the inducible nature of the AmpC-type β-lactamase encoded by the *blaDHA* gene [24], which can achieve high-level expression through mutation. Overexpression of this enzyme can lead to resistance to broad-spectrum cephalosporins, including cefotaxime, ceftazidime, and ceftriaxone [25].The AmpC-type β-lactamase exhibits activity against penicillins but possesses even higher activity against cephalosporins [25]. When strongly induced, it can generate large quantities of the AmpC enzyme that hydrolyze cephalosporins, resulting in a resistant phenotype. However, penicillin drugs are poor inducers. The amount of enzyme produced under their induction is insufficient to hydrolyze the drug effectively, thus the bacterium presents as susceptible. Within the class 2/1 hybrid integron carried by SH-8, namely *sul1*-*qacEΔ1*-*aadA1*-*dfrA1*-*lnuF*, 5 different resistance genes are arranged in tandem. Driven by the integron promoter (Pc), these distinct resistance genes are coordinately expressed. For instance, *sul1* and *dfrA1* target different enzymes in the bacterial folate synthesis pathway, conferring resistance to sulfonamides and trimethoprim, respectively. The efflux pump encoded by *qacEΔ1* acts synergistically with modifying enzymes encoded by genes such as *aadA1* and *lnuF*. The efflux pump reduces drug influx into the cell, while the modifying enzymes chemically inactivate any drugs that enter. This dual strategy of prevention and neutralization enhances bacterial resistance to aminoglycosides, lincosamides, and other drug classes, forming a highly effective MDR defense system. The transposable unit harboring this integron can transpose (mediated by transposase and associated elements) between locations on the bacterial chromosome and plasmids. It can also disseminate between different bacterial cells. When bacteria carrying this transposable unit encounter other bacteria (e.g., within microbial communities in the gut or soil), the unit can facilitate the transfer of the integron and its MDR gene cassette array to recipient cells via horizontal gene transfer (HGT) mechanisms such as conjugation or transformation. This enables recipient bacteria to rapidly acquire the MDR phenotype, accelerates the spread of resistance genes within bacterial populations, and exacerbates the challenges of clinical anti-infective therapy. In addition to the resistance genes located within integrons, we identified the *msr*(*E*)*-mph*(*E*) operon downstream on the chromosome of strain SH-8. The *msr*(*E*) and *mph*(*E*) genes confer high-level resistance to macrolide antibiotics. Specifically, the *msr*(*E*) gene encodes an ABC transporter protein that actively pumps macrolide antibiotics out of the cell via an efflux mechanism [26]; whereas the *mph*(*E*) gene encodes a macrolide 2′-phosphotransferase that inactivates the antibiotic through chemical modification (phosphorylation) [27]. Together, these two mechanisms form a unique resistance system. The *msr*(*E*)-*mph*(*E*) operon is widely distributed among various bacterial species, such as *Acinetobacter baumannii* [28] and *Pasteurella multocida* [29], and has also been detected in *P. mirabilis*. Although studies on the role of the *msr*(*E*)*-mph*(*E*) operon in *P. mirabilis* are still limited, research by Blackwell GA et al. [28] on *Acinetobacter baumannii* revealed that the *msr*(*E*)*-mph*(*E*) operon can function as an independent mobile genetic module. This module is flanked by specific pdif sites (inverted sequences recognized by the XerC-XerD recombinase), enabling its potential mobility between different DNA molecules—such as plasmids and chromosomes—in various bacteria. The presence of the *msr*(*E*)*-mph*(*E*) operon enhances the macrolide resistance capability of *P. mirabilis*. Coupled with its potential mobility, this operon may pose challenges for clinical treatment and contribute to the spread of antimicrobial resistance. The SH-8 contains five different IS (IS1006, ISVsa3, ISAba1, IS6, and IS26). It has been reported that IS26 can promote the fluoroquinolone resistance gene *cfr* [30] and plasmid-mediated quinolone resistance genes, while ESBL genes [31] can facilitate the horizontal gene transfer of resistance elements. The results of this study indicate that an efficient antibiotic resistance gene transmission network, composed of integrons, transposons, and insertion sequences, has formed a unique mechanism in zoonotic pathogens. In particular, the co-occurrence of IS26 and the class 2 integron may significantly accelerate the flow and spread of MDR gene cassettes between bacterial chromosomes and plasmids, potentially through mediating gene rearrangements and forming composite transposons.

The heatmap revealed a high degree of similarity in the ARGs carried by SH-8 and strains from other sources, which is likely attributable to horizontal gene transfer. Furthermore, the resistance gene profiles of human-derived and animal-derived *P. mirabilis* strains also showed considerable overlap. This indicates that drug resistance genes in *P*. *mirabilis* can be transmitted between animals and humans via the food chain, environmental exposure, or direct contact. This not only poses public health risks but also facilitates the spread of different resistance genotypes within the *P. mirabilis* population, thereby complicating clinical treatment.

Horizontal transfer of ARGs enables bacteria to rapidly acquire multiple resistance mechanisms, creating MDR or even extensively drug-resistant (XDR/pandrug-resistant) strains. These resistance genes are disseminated between different bacterial species, genera, and even across diverse microbial species via mobile genetic elements (MGEs) such as plasmids, transposons, and integrons. The emergence of MDR strains not only complicates treatment but also exacerbates these challenges. Through antimicrobial susceptibility testing analysis of *P. mirabilis* SH-8, it was found that this strain is sensitive to penicillin antibiotics. Therefore, some penicillin drugs approved for veterinary use (such as penicillin G and amoxicillin) can be selected as treatment options for porcine-origin *P. mirabilis*.

## 5. Conclusions

As an important opportunistic pathogen, *P. mirabilis* has become a growing public health concern due to increasing antibiotic resistance. This study systematically investigated the AMR patterns and underlying mechanisms in *P. mirabilis*, providing critical insights for controlling and treating infections caused by this pathogen.

## Figures and Tables

**Table 1:** The antimicrobial susceptibility testing results for *P. mirabilis*.

Antimicrobial Classes	Antimicrobial Agent	MIC of SH-8 (μg/mL)	The Breakpoint of MIC (μg/mL)	Result	Resistance Genes
penicillins	amoxicillin ampicillin	1 1	≥32 ≥32	S S	*blaDHA-1*
cephalosporins	cefazolin ceftriaxone	64 4	≥32 ≥4	R R	*blaDHA-1*
monobactams	aztreonam	0.06	≥16	S	-
aminoglycosides	gentamicin	8	≥8	R	*aac*(*3*)*-IVa*, *aac*(*6′*)*-Ib10*
macrolides	azithromycin	256	≥32	R	*mphE*, *msrE*, *blaCRP*
phenicols	chloramphenicol	0.5	≥32	S	*catA4*
sulfonamides	trimethoprim	512	≥16	R	*dfrA1*, *sul1*

The abbreviations R and S denote resistant and susceptible, respectively.

## Data Availability

The original contributions presented in this study are included in the article and Supplementary Materials. Further inquiries can be directed to the corresponding authors.

## Supplementary Materials

The following supporting information can be downloaded at: https://www.mdpi.com/article/10.3390/vetsci12121113/s1, xlsx S1: Detailed information of the strains used to generate heat maps.
